# AMIGO2 contained in cancer cell-derived extracellular vesicles enhances the adhesion of liver endothelial cells to cancer cells

**DOI:** 10.1038/s41598-021-04662-1

**Published:** 2022-01-17

**Authors:** Runa Izutsu, Mitsuhiko Osaki, Hideyuki Nemoto, Maho Jingu, Ryo Sasaki, Yusuke Yoshioka, Takahiro Ochiya, Futoshi Okada

**Affiliations:** 1 Division of Experimental Pathology, Faculty of Medicine, Tottori University, 86 Nishi-cho, Yonago, Tottori 683-8503 Japan; 2 Chromosomal Engineering Research Center, Tottori University, Yonago, Tottori, Japan; 3 Department of Molecular and Cellular Medicine, Institute of Medical Science, Tokyo Medical University, Tokyo, Japan

**Keywords:** Cancer, Pathogenesis

## Abstract

Adhesion of cancer cells to vascular endothelial cells in target organs is an initial step in cancer metastasis. Our previous studies revealed that amphoterin-induced gene and open reading frame 2 (AMIGO2) promotes the adhesion of tumor cells to liver endothelial cells, followed by the formation of liver metastasis in a mouse model. However, the precise mechanism underlying AMIGO2-promoted the adhesion of tumor cells and liver endothelial cells remains unknown. This study was conducted to explore the role of cancer cell-derived AMIGO2-containing extracellular vesicles (EVs) in the adhesion of cancer cells to human hepatic sinusoidal endothelial cells (HHSECs). Western blotting indicated that AMIGO2 was present in EVs from AMIGO2-overexpressing MKN-28 gastric cancer cells. The efficiency of EV incorporation into HHSECs was independent of the AMIGO2 content in EVs. When EV-derived AMIGO2 was internalized in HHSECs, it significantly enhanced the adhesion of HHSECs to gastric (MKN-28 and MKN-74) and colorectal cancer cells (SW480), all of which lacked AMIGO2 expression. Thus, we identified a novel mechanism by which EV-derived AMIGO2 released from AMIGO2-expressing cancer cells stimulates endothelial cell adhesion to different cancer cells for the initiate step of liver metastasis.

## Introduction

Metastasis is associated with more than 90% of cancer-related deaths^1^, with the liver being the most frequent site (59%) of distant metastases^2^. Distant metastasis is initiated via the adherence of cancer cells to vascular endothelial cells of the target organ. It has been shown that preferential binding between adhesion molecules, such as integrin α4β1 and vascular cell adhesion molecule 1 (VCAM-1), enhances the attachment between cancer cells and endothelial cells^3^. However, the precise mechanism underlying the adhesion of cancer cells to endothelial cells is not fully understood. Hence, there is an urgent need to comprehensively elucidate the mechanism underlying this adhesion in order to prevent and treat cancer metastasis.

Amphoterin-induced gene and open reading frame 2 (AMIGO2) is a member of the AMIGO protein family, which have been identified as novel cell adhesion molecules^4^. In our previous study, we revealed that AMIGO2 regulates the development of liver metastasis in a mouse model^5^. The LV12 cell subline, which exhibits high expression of AMIGO2 and enhances metastasis to the liver, was established using QRsP-11 mouse fibrosarcoma cells through an in vivo selection procedure. It has been demonstrated that the overexpression of AMIGO2 in parental QRsP-11 cells enhanced the adhesion between liver endothelial cells and tumor cells, whereas AMIGO2 knockdown in LV12 cells attenuated their attachment to endothelial cells, and consequently, liver metastasis^5^. These observations suggest a correlation between the expression of AMIGO2 in cancer cells and their adhesion to liver endothelial cells. In fact, our histopathological study revealed a close association between AMIGO2 expression in primary tumors from patients with colorectal cancer and liver metastasis^6^. In addition, high AMIGO2 mRNA has been reported to be positively associated with disease-free survival and reduced overall survival in patients with gastric cancer^7^. Thus, AMIGO2 expression in cancer cells may enhance the adhesion of cancer cells to liver endothelial cells, thereby facilitating the formation of liver metastasis. Based on this hypothesis, we postulated two possible mechanisms for enhancing such cell adhesion by AMIGO2. One is that AMIGO2 expressed in cancer cells enhances the adhesiveness of the cancer cells themselves. The other is that AMIGO2-containing extracellular vesicles (EVs) released from AMIGO2-overexpressing cancer cells may stimulate to enhance the adhesiveness of endothelial cells to cancer cells.

EVs contain proteins, nucleic acids, lipids, amino acids, and metabolites, which generally reflect the cell of origin^8–10^. Recent studies have reported that EVs secreted from cancer cells are incorporated into the endothelial cells of target organs and induce the establishment of a pre-metastatic niche^11,12^. Further, cancer cell-derived EVs have been shown to disrupt the integrity of endothelial cells^13,14^ and promote vascular angiogenesis^15,16^. However, the mechanism underlying the adhesion between endothelial and cancer cell has not yet been elucidated.

The present study is the first to focus on the promotion of the above-described adhesion by cancer cell-derived EVs during establishment of liver metastasis formation. We found that AMIGO2, a liver metastasis driver molecule, was transferred from human gastric cancer cells to hepatic sinusoidal endothelial cells via tumor-derived EVs, and that the AMIGO2 delivered by EVs enhanced the adhesion of endothelial cells to cancer cells.

## Results

### AMIGO2 was present in EVs derived from AMIGO2-overexpressing MKN-28 cells

**Figure 1:**
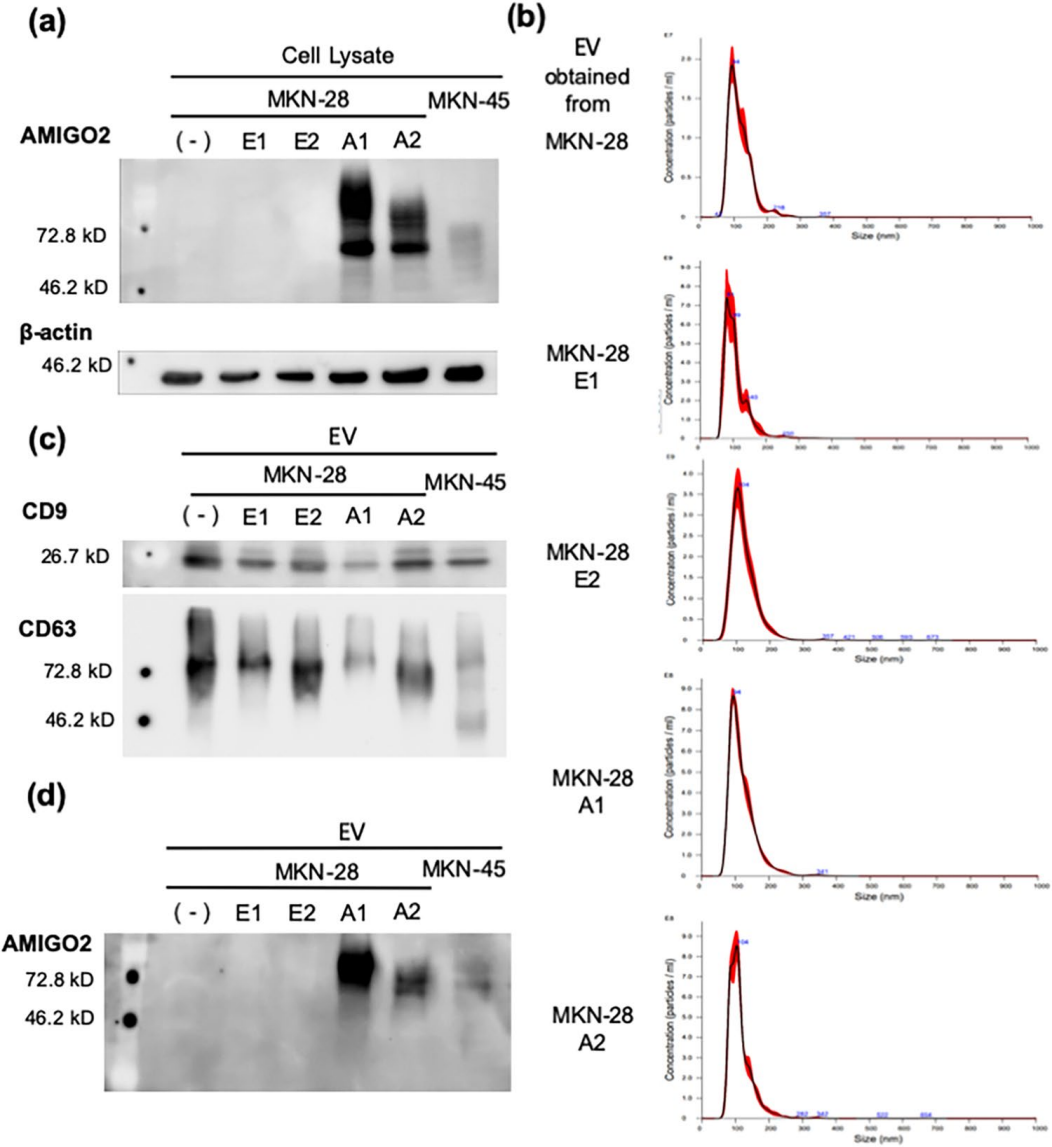
AMIGO2 was present in EVs derived from AMIGO2-overexpressing MKN-28 cells. (**a**) AMIGO2 was only detected in AMIGO2-overexpressing cells (MKN-28 A1 and A2) and MKN-45, as determined by western blotting. Full-length blots were presented in Supplementary Fig. S1. (**b**) EV size was determined via nanoparticle tracking analysis. (**c**) CD9 and CD63 expression was detected by western blotting. Full-length blots were presented in Supplementary Fig. S2. (**d**) AMIGO2 present in EVs obtained from AMIGO2-overexpressing cells (A1 and A2) and MKN-45 cells, as determined by western blotting. Full-length blots were presented in Supplementary Fig. S3.

Our previous study showed that the expression of AMIGO2 in tumor cells that rarely metastasize to the liver enhanced the adhesion between liver endothelial cells and liver metastases. However, the mechanism by which AMIGO2 from tumor-derived EVs mediates the adhesion between tumor and endothelial cells has not been clarified. To investigate the function of AMIGO2 in EVs derived from AMIGO2-overexpressing cancer cells, we established AMIGO2-overexpressing MKN-28 human gastric cancer cell lines (MKN-28 A1, A2) and control vector-transfected cell lines (MKN-28 E1, E2). MKN-45 cells expressed high levels of AMIGO2 and were used as a positive control in western blots. AMIGO2 was expressed in MKN-28 A1, A2, and MKN-45 cells (Fig. 1a). EVs were isolated from the supernatant of MKN-28 cells, their transfectants, and MKN-45 cells via ultracentrifugation. The size of the collected EVs was determined using nanoparticle tracking analysis (Fig. 1b). The mean diameters of the EVs secreted from MKN-28, MKN-28 E1, MKN-28 E2, MKN-28 A1, and MKN-28 A2 cells were 116.9 ± 6.00 nm, 120.78 ± 7.39 nm, 121.65 ± 1.78 nm, 119.76 ± 9.56 nm, and 109.76 ± 3.50 nm, respectively, with no significant difference in size compared to those of EVs obtained from MKN-28 parental cells. Moreover, representative EV markers CD9 and CD63 were detected in EVs from all cells, confirming the former as EVs (Fig. 1c). To assess whether AMIGO2-overexpressing cancer cells released AMIGO2-containing EVs, we performed western blotting to measure the EV-derived AMIGO2 protein levels. AMIGO2 was detected in EVs derived from AMIGO2-transfectanted and MKN-45 cells (Fig. 1d). In contrast, EVs from parental cells and control vector-transfected cells did not contain AMIGO2 protein. These data confirmed that AMIGO2-overexpressing cancer cells released AMIGO2-containing EVs.

### AMIGO2 was incorporated to human hepatic sinusoidal endothelial cells (HHSECs) via EVs

**Figure 2:**
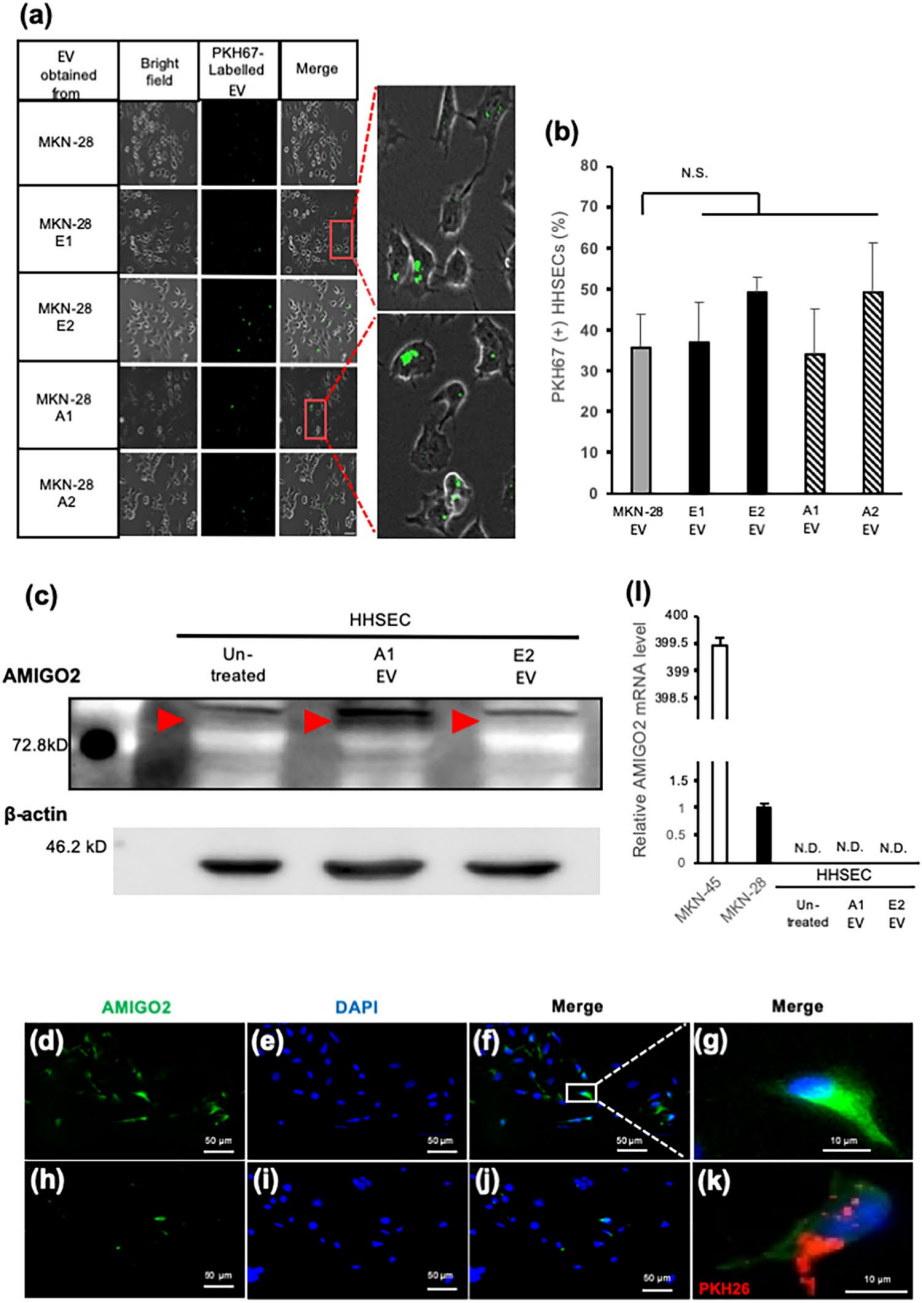
AMIGO2 was delivered to human hepatic sinusoidal endothelial cells. (**a**) EVs (green) were observed to incorporate into HHSECs within 6 h by microscopy (Keyence, Japan). Scale bar, 50 µm. (**b**) EVs from each cell line were almost evenly incorporated into HHSECs. Bar graphs show mean ± SD (n = 5 in each group). N.S., no significance based on the Dunnett's test. (**c**) The expression of AMIGO2 in HHSECs treated with AMIGO2-containing EVs was increased by western blotting. Full-length blots were presented in Supplementary Fig. S4. (**d**–**k**) AMIGO2 (green) in HHSECs was detected by immunocytochemistry. HHSECs were treated with A1 EVs (**d**–**g**,**k**) or E2 EVs (**h**–**j**). (**k**) AMIGO2 was transferred into HHSECs via A1 EVs (red). Unmerged data were shown in Supplementary Fig. S9. (**l**) The level of AMIGO2 mRNA did not increase in HHSECs treated with AMIGO2-containing EVs by quantitative polymerase chain reaction. Bar graphs show mean ± SD (n = 3 in each group). N.D., not detected.

An EV uptake assay was performed to investigate whether HHSECs incorporated the EVs obtained from MKN-28 cells and their transfectants. EVs were labeled with PKH67 fluorescent dye and incubated with the HHSECs. As expected, the EVs were internalized by HHSECs within 6 h (Fig. 2a). PKH67-labeled EVs from each cell line were almost equally incorporated into HHSECs (Fig. 2b). These results indicated that EV uptake efficiency was independent of the presence or absence of AMIGO2 in EVs. Next, we measured AMIGO2 expression in HHSECs that internalized AMIGO2-containing EVs. Though HHSECs did not express AMIGO2 prior to EV treatment, AMIGO2 expression was observed following the addition of AMIGO2-containing EVs (Fig. 2c). Relative levels of AMIGO2 protein were quantified and shown in Supplementary Fig. S5. Moreover, immunocytochemistry revealed that AMIGO2 was mainly localized in the cytoplasm of HHSECs treated with AMIGO2-containing EVs (Fig. 2d–g,k). Moreover, immunocytochemistry revealed that AMIGO2 was mainly localized in the cytoplasm of HHSECs treated with AMIGO2-containing EVs (Fig. 2d–g,k). No fluorescence was detected in HHSEC treated with EV lacking AMIGO2 (Fig. 2h–j). Figure 2k showed that EVs (red) and AMIGO2 (green) were colocalized in PKH26 labelled A1 EVs treated HHSECs, indicating that AMIGO2 protein was transferred to HHSECs by EVs. Treatment of EVs did not induce the expression of AMIGO2 mRNA in HHSECs (Fig. 2l). These data indicate that AMIGO2 protein is delivered to HHSECs via EVs, without the induction of endogenous AMIGO2 expression.

### Incorporation of AMIGO2 via EVs into HHSECs promoted their adhesion to cancer cells

**Figure 3:**
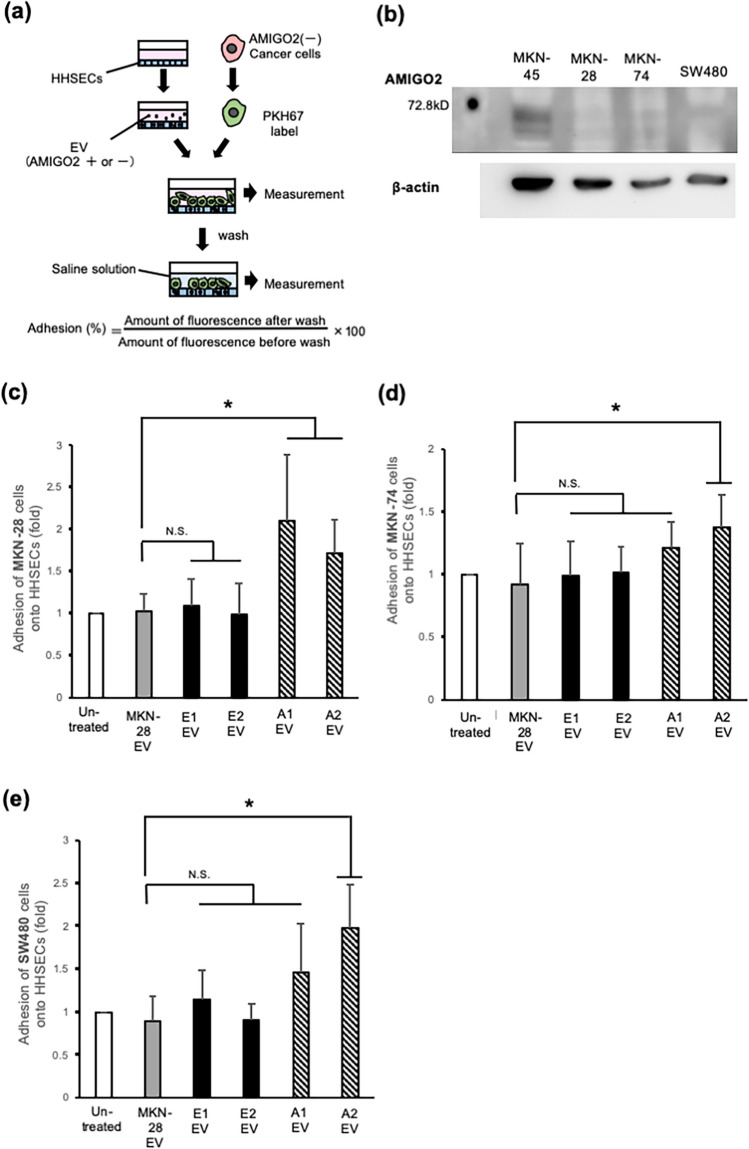
Incorporation of AMIGO2 into HHSECs via EVs mediates adhesion to cancer cells. (**a**) Schematic describing the cancer cell-HHSEC adhesion assay. (**b**) MKN-28, MKN-74, and SW480 cells did not express AMIGO2 by western blotting. Full-length blots were presented in Supplementary Fig. S6. (**c**) The relative adhesion of MKN-28 cells to HHSECs in adhesion assay. Bar graphs show mean ± SD (n = 12 in each group). *P < 0.05 (Dunnett's test). (**d**) The relative adhesion of MKN-74 cells to HHSECs in adhesion assay. Bar graphs show mean ± SD (n = 19 in each group). *P < 0.05 (Dunnett's test). (**e**) The relative adhesion of SW480 cells to HHSECs in adhesion assay. Bar graphs show mean ± SD (n = 11 in each group). *P < 0.05 (Dunnett's test). N.S., no significance based on the Dunnett's test. These experiments were performed at least 3 times.

To determine whether AMIGO2-containing EVs enhance the adhesion of HHSECs to cancer cells, a cancer cell-HHSEC adhesion assay was performed (Fig. 3a). HHSECs were treated with AMIGO2-containing EVs for 6 h. After EVs treatment, PKH67-labeled cancer cells were seeded onto HHSECs. Human gastric cancer cell lines MKN-28 and MKN-74 and the human colorectal cancer cell line SW480 were used in this assay. These three cell lines did not express AMIGO2 (Fig. 3b). When HHSECs were treated with AMIGO2-containing EVs secreted by MKN-28 A1 and A2 cells, their adhesion to MKN-28 cells was significantly higher than that of HHSECs treated with AMIGO2-lacking EVs obtained from MKN-28, MKN-28 E1, and E2 cells (Fig. 3c). Moreover, we examined the adhesion of HHSECs to MKN-74, another human gastric cancer cell line, and observed that adhesion increased following treatment with MKN-28 A1- and A2-derived EVs (Fig. 3d). Of note, adhesion to SW480 was also increased upon treatment with AMIGO2-containing EVs obtained from MKN-28 A1 and A2 cells (Fig. 3e). The enhanced adhesion of HHSECs to MKN-74 and SW480 cells following EV-derived AMIGO2 from MKN-28 A1 and A2 cells suggested that AMIGO2-containing EVs may promote the adhesion to cancer cell lines from different tissues, such as colorectal and gastric cancers. We examined that the adhesion of MKN-28 to HHSECs transiently transfected with the AMIGO2 plasmids was significantly increased compared to that of HHSECs transfected with the empty plasmids. This data was shown in Supplementary Fig. S8. Taken together, cancer cell-derived EVs containing AMIGO2 modulates the function of liver endothelial cells, enhancing their adhesion to cancer cells.

### EV-derived AMIGO2 did not affect HHSEC proliferation and migration

**Figure 4:**
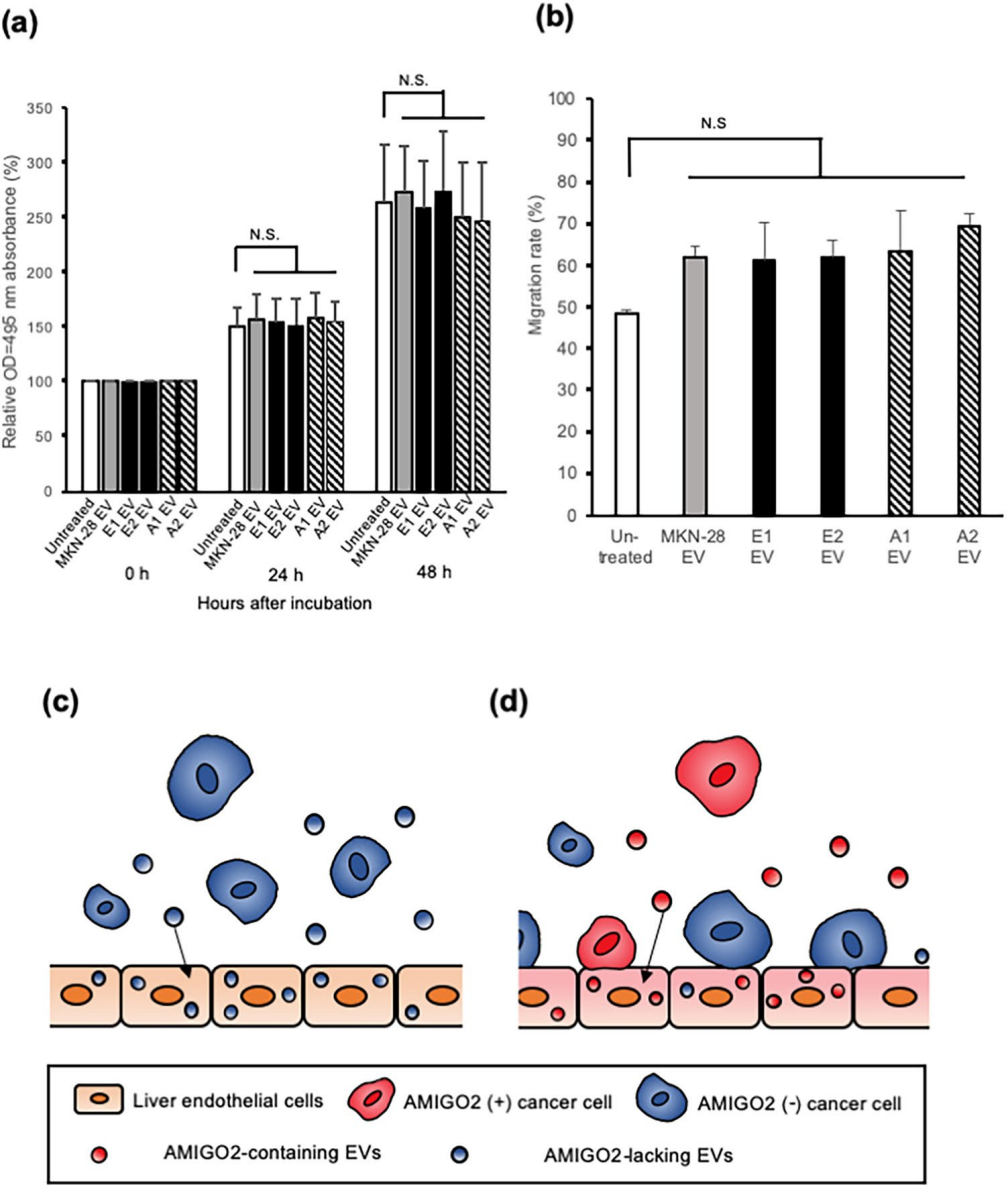
EVs-derived AMIGO2 did not affect HHSEC proliferation and migration. (**a**) HHSEC proliferation was evaluated using crystal violet assay. Bar graphs show mean ± SD (n = 10 in each group). This experiment was performed at 2 times. N.S., no significance based on the Dunnett's test. (**b**) Wound-healing assays were performed to evaluate the migration of HHSECs. Bar graphs show mean ± SD (n = 5 in each group). This experiment was performed at 3 times. Typical data were presented in Supplementary Fig. S7. *P < 0.05 (Dunnett's test). N.S., no significance on the Dunnett's test. (**c**) AMIGO2(-) EVs incorporated into liver endothelial cells do not enhance their adhesion to cancer cells. (**d**) When AMIGO2(+) EVs are incorporated into liver endothelial cells, AMIGO2(+) cancer cells as well as AMIGO2(-) cancer cells adhere to the endothelial cells. AMIGO2(-), AMIGO2-dificient cells; AMIGO2(+), AMIGO2-containing cells.

To further investigate the function of EV-derived AMIGO2, the proliferation and migration of HHSECs treated with AMIGO2-containing EVs were evaluated. First, the proliferation of EV-treated HHSECs was analyzed via a crystal violet assay. AMIGO2-containing EVs did not affect the proliferation of HHSECs (Fig. 4a). To evaluate the migration ability of HHSECs after EVs treatment, a wound-healing assay was performed. Notably, the migration of HHSECs was not associated with EV-derived AMIGO2 (Fig. 4b), indicating that AMIGO2 was not implicated in the promotion of HHSEC migration and proliferation.

## Discussion

Recent studies have proposed that intercellular communication via cancer cell-derived EVs plays an important role in the proliferation, migration, invasion, and stemness maintenance of cancer cells as well as in the propensity of endothelial cells to establish distant metastasis^13–21^. While the adhesion between cancer and endothelial cells has been established as a critical step in metastasis, the effects of EVs on cell adhesion have remained unclear. In this study, we demonstrated for the first time that AMIGO2-containing EVs derived from AMIGO2-expressing cancer cells were treated with liver endothelial cells, significantly enhancing their adhesion to cancer cells. This effect may be of major relevance to the establishment of a pre-metastasis niche in the sinusoid, an event that is necessary for the initiation of liver metastasis.

We showed that AMIGO2-containing EVs derived from AMIGO2-expressing gastric cancer cells (MKN-28 A1 & A2) enhanced the adhesion of HHSECs to the same gastric cancer cells (MKN-28) as well as to different gastric cancer cells (MKN-74) and colorectal cancer cells (SW480) (Fig. 3c–e). These results indicated that the AMIGO2 present in EVs promoted the adhesion of HHSECs to cancer cells, regardless of origin of the cancer cells. Several studies have reported that cell proliferation, survival, and invasion are driven by intratumor heterogeneity during primary tumor growth^22,23^. In addition, various cellular components present in circulating tumor cells, such as DNA, RNA, proteins, and metabolites, influence the heterogeneity of metastatic tumors^24^. We propose a novel mechanism by which AMIGO2-expressing cancer cells within tumor tissues stimulate endothelial cells in target organs for enabling the establishment of pre-metastatic niches via the effect of AMIGO2-containing EVs, which enhance the adhesion of endothelial cells to not only AMIGO2-positive but also AMIGO2-negative cancer cells (Fig. 4c,d). The phenomenon where cancer cells that do not express AMIGO2 adhere to HHSECs treated with AMIGO2-containing EVs is a prime example of metastatic cancer cell heterogeneity.

The present study demonstrated that HHSECs incorporated AMIGO2 proteins, and as a result, exhibited enhanced adhesion to cancer cells. However, the mechanism by which EV-derived AMIGO2 enhances the adhesion of endothelial cells to cancer cells remains unknown. Two possible mechanisms were considered. First, AMIGO2 delivered into HHSECs was transported to the plasma membrane where it functions as an intercellular adhesion molecule that can bind to cancer cells. Members of the AMIGO family, including AMIGO1, AMIGO2, and AMIGO3, are known to bind homophilically or heterophilically^4^. Thus, it is possible that the delivered AMIGO2 localizes to the cell membrane of HHSECs where it promotes adhesion to cancer cells (Fig. 3b). This would suggest the binding of AMIGO2 present on the surface of HHSECs to AMIGO family proteins or other adhesion molecules expressed on cancer cells (blue cells in Fig. 4d). However, we did not observe the strong localization of AMIGO2 in the plasma membrane of HHSECs. In the second scenario considered, EV-derived AMIGO2 would facilitate the adhesion of HHSECs by activating and/or upregulating other adhesion molecules. In human umbilical vein endothelial cells (HUVECs), the expression of AMIGO2 upregulated the expression of the cell–cell adhesion molecule E-selectin^25–29^. Several studies have reported that the cell surface molecules (Sialyl-Lewis A/X and integrin α4β1) on cancer cells and adhesion molecules (E-selectin and VCAM-1) on the surface of liver endothelial cells promote liver metastasis through specific binding^3,27,30–32^. Further studies should determine the expression of adhesion molecules on the surface of HHSECs treated with AMIGO2-containing EVs by proteomic analysis.

In conclusion, our study revealed that cancer cell-derived EVs containing AMIGO2 enhanced the adhesion of liver endothelial cells to cancer cells. Therefore, AMIGO2 containing EVs represents a novel therapeutic target for preventing the liver metastasis of human cancer cells.

## Methods

### Cell lines and culture conditions

Three human gastric cancer cell lines (MKN-28, MKN-45, and MKN-74), a human colorectal cancer cell line (SW480), and HHSECs (human hepatic sinusoidal endothelial cells) were used in this study. MKN-28 and its transfectants, MKN-45 and MKN-74, were maintained in Dulbecco’s modified Eagle medium (DMEM; 05919, Nissui Pharmaceuticals, Tokyo, Japan) supplemented with 10% fetal bovine serum (FBS; 1370978, Gibco, Gaithersburg, MD, USA), P.S. (100 Units/mL penicillin and 100 µg/mL streptomycin, Gibco), and L-glutamine (30 mg/mL, Nacalai Tesque, Kyoto, Japan). SW480 cells were cultured in Roswell Park Memorial Institute 1640 medium (13485; Gibco) supplemented with 10% FBS. HHSECs were maintained in endothelial cell medium (1001; ScienCell) in bovine plasma fibronectin (10%; 8284; ScienCell)-coated dishes. All cell lines were cultured at 37 °C in an atmosphere of 95% air and 5% CO_2_.

### Plasmid transfection

Human AMIGO2 expression vector (pEZ-M02/AMIGO2) and control vector (pEZ-M02) were obtained from GeneCopeia (Rockville, MD, USA). These plasmids were transfected into MKN-28, an AMIGO2-lacking cell line, in order to establish stable AMIGO2-overexpressing MKN-28 cell lines (MKN-28 A1 and A2) and control lines (MKN-28 E1 and E2), respectively. MKN-28 cells were transfected with 1 µg pEZ-M02/AMIGO2 or its corresponding negative control (pEZ-M02) using Lipofectamine 2000 (Invitrogen; Thermo Fisher Scientific, Waltham, MA, USA) as per the manufacturer's protocol. Transfectants stably expressing the introduced vector plasmid were selected by continuous neomycin treatment at 450 µg/mL (10131035, Thermo Fisher Scientific). Neomycin-resistant cells were cloned by the limiting dilution method and maintained in a medium containing neomycin.

### Isolation of EVs

MKN-28 cells and transfectants were cultured until they reached 80% confluency in 10% FBS DMEM; the medium was then replaced with advanced DMEM without serum supplements (12491-015; Gibco) containing L-glutamine. After 48 h, the supernatant was collected and centrifuged at 2000 × *g* for 10 min at 4 °C to remove cells and cell debris. The supernatant was filtered through a 0.22 µm membrane filter (Millipore, Billerica, MA, USA). The filtered supernatant was centrifuged at 210,000 × *g* for 70 min at 4 °C using a Beckman Coulter Optima XPN-80 ultracentrifuge with an SW41Ti rotor. After centrifugation, the supernatant was removed, and the pellet was resuspended in phosphate-buffered saline (PBS) centrifuged at 210,000 × *g* for 70 min at 4 °C. Subsequently, PBS was removed, and the pellet containing the EVs was resuspended in PBS. The protein concentration in the EVs was measured using a Micro BCA Protein Assay Kit (23235; Thermo Fisher Scientific). The number of EVs was measured using NanoSight NS300 (Malvern Panalytical, Malvern, England) at camera setting 16 and threshold setting of 8. Videos were recorded five times per sample for 1 min and analyzed using nanoparticle tracking analysis software (version 3.1, Malvern Panalytical).

### Real-time polymerase chain reaction (PCR)

Total RNA was extracted from cells using TRIzol reagent (15596026; Thermo Fisher Scientific), and 1 μg of total RNA was reverse transcribed into cDNA using the TaKaRa PrimeScript RT master mix (TaKaRa Bio, Otsu, Japan). Five microliter of cDNA was used for performing quantitative PCR. Quantitative PCR was performed on a Roche LightCycler 480 (Roche Diagnostics, Palo Alto, CA, USA) using SYBR Premix Ex Taq II (Tli RNaseH Plus; TaKaRa Bio). The cycling program involved heating at 50 °C for 2 min, 95 °C for 10 min, followed by 35 cycles at 95 °C for 15 s 60 °C for 1 min. β-actin was used as the internal control. The following primers were used: AMIGO2-F: 5′-CCCCTGCAAGTGTAAAACCA-3′, AMIGO2-R: 5′-AGGGGTTCCAAAAACACCAC-3′; β-actin-F: 5′-AGAGGGAAATCGTGCGTGAC-3′, β-actin-R: 5′-CAATAGTGACCTGGCCGT-3′.

### Western blotting

Cells were lysed in ice-cold lysis buffer (1% NP-40, 50 mM Tris (pH 7.5), 165 mM NaCl, 10 mM EGTA, 1 mM Na_3_VO_4_, 10 mM NaF, 1 mM PMSF, 10 μg/mL aprotinin, and 10 μg/mL leupeptin). Proteins were separated on 10% (CD63 and AMIGO2) or 12% (CD9) sodium dodecyl sulfate–polyacrylamide gels and transferred onto polyvinylidene fluoride membranes (ISEQ00010; Merck Millipore, Darmstadt, Germany). The membranes were blocked using 2.5% skim milk (prepared in T-PBS) at room temperature (RT) for 2 h. The membranes were then probed with the following primary antibodies: rat monoclonal anti-AMIGO2 antibody (1:10,000; rTNK1b012a)^33^, mouse monoclonal anti-β-actin antibody (1:5,000; A5441; Sigma Aldrich, St. Louis, MO, USA), mouse monoclonal anti-CD9 antibody (1:2,500; 12A12; Cosmo Bio, Tokyo, Japan), or mouse monoclonal anti-CD63 antibody (1:5,000; 8A12; Cosmo Bio) overnight at 4 °C. The membranes were then incubated with a goat polyclonal anti-rat IgG horseradish peroxidase-conjugated antibody (1:10,000; ab98425; Abcam, Cambridge, United Kingdom) or a goat polyclonal anti-mouse IgG antibody (1:5,000; PM009-7; MBL, Nagoya, Japan) at RT for 20 min. Protein signals were detected using an enhanced chemiluminescence kit (RPN2232; GE Healthcare; Buckinghamshire, United Kingdom) and analyzed using ChemiDoc Touch MP (Bio-Rad, Hercules, CA, USA).

### Incorporation of EVs into HHSECs

HHSECs (3 × 10^4^ cells) were seeded in 24-well plates (3526, Corning, Corning NY, USA), incubated for 24 h, and then treated with 5 µg of EVs at 37 °C. EVs were labeled using a PKH67 green fluorescent kit (MIDI67-1KT; Sigma Aldrich). EVs were incubated with 4 µM PKH67 for 6 min at RT and then washed with PBS to remove free PKH67 via centrifugation at 14,000 × *g* for 2 min (five washes). After 6 h, EVs incorporated into HHSECs were observed under a fluorescence microscope (BZ-X710; Keyence, Osaka, Japan) and the number of PKH67-positive HHSECs was counted based on the images taken by microscope (Keyence, Japan).

### Immunofluorescence

HHSECs (3 × 10^4^ cells) were seeded in Lab-Tek 8-well chamber slides (177402; Nunc, Rochester, NY, USA) and incubated for 48 h. Next, cells were treated with EVs (10 µg/well) for 6 h. EVs were labeled using a PKH26 red fluorescent kit (MINI26-1KT; Sigma Aldrich). HHSECs were then fixed with 4% paraformaldehyde (163-20145; Wako, Osaka, Japan) and treated with 10% normal goat serum (426042; Nichirei, Tokyo, Japan). Then, HHSECs were probed overnight with the primary antibody, i.e., anti-AMIGO2 (1:1000, rTNK1b012a) at 4 °C, followed by incubation with anti-rat IgG H&L Alexa Fluor 488 (1:5000; ab150157; Abcam). Nuclei were stained with 4′,6-diamidino-2-phenylindole (340-07971; Dojindo, Kumamoto, Japan).

### Cancer cell-endothelial cell adhesion assay

The cancer cell-HHSEC adhesion assay was performed in accordance to a previously reported method with slight modifications^5^. Briefly, a 96-well plate (165305; Thermo Fisher Scientific) was coated with fibronectin for 2 h. A total of 3 × 10^4^ HHSECs were seeded per well, cultured for 72 h, and then treated with 1.5 µg EVs (1.25 × 10^–5^ µg per cell) for 6 h. Cancer cells (2 × 10^5^) labeled with PKH67 green fluorescent dye (PKH67GL-1KT, Sigma Aldrich) were then plated onto HHSECs and further cultured for 30 min. Non-adherent cancer cells were removed by washing with saline solution (1326; Otsuka, Tokyo, Japan), and the adherent cancer cells were quantified using a fluorescence plate reader (Infinite M200 PRO; Tecan, Männedorf, Switzerland) at an excitation wavelength of 485 nm and an emission wavelength of 535 nm. The percentage of adherence was calculated as the fluorescence ratio, i.e., (post-wash fluorescence/pre-wash fluorescence) × 100.

### Cell proliferation assay

HHSECs (3 × 10^3^) were seeded in 96-well plates (3595; Corning) and incubated for 24 h, after which they were treated with 0.075 µg EVs (1.25 × 10^−5^ µg per cell). After 24 and 48 h of incubation, HHSECs were fixed with 5% glutaraldehyde, dried and stained using 0.5% crystal violet for 5 min. After washing with running water, cells were left to air dry. Crystal violet was eluted using 10% acetic acid and quantified by measuring the absorbance at 495 nm using a fluorescence plate reader (Infinite M200 PRO, Tecan).

### Wound-healing assay

A wound-healing assay was performed to measure cell migration activity as described by Taniguchi et al.^34^. HHSECs (3 × 10^4^) were seeded in 24-well plates (3526; Corning) and incubated for 5 days until the cells formed a monolayer. Wounds were created by scratching the cells with 200 µL pipette tips, and the medium containing the non-adherent cells was removed. At 0 h and 15 h after treatment of HHSECs with 7.5 µg EVs (1.25 × 10^–5^ µg per cell), the scratched area was observed using a phase-contrast microscope (BZ-X710, Keyence). Cell migration was determined as the rate of cells moving to the scratched area, which was quantified using ImageJ (version 1.53, https://imagej.nih.gov/ij/, National Institutes of Health, Bethesda, MD, USA).

### Statistical analyses

All data are presented as the mean ± standard deviation. The significance of the differences was assessed using the Student's t-test and Dunnett's test. *P* < 0.05 was considered significant.

## Supplementary Information

Supplementary Information.
