# Supplementary material for: AMIGO2 contained in cancer cell-derived extracellular vesicles enhances the adhesion of liver endothelial cells to cancer cells
Source: Sci Rep. 2022 Jan 17;12:792. doi: 10.1038/s41598-021-04662-1 (PMC8763894; doi:10.1038/s41598-021-04662-1)
Supplement: Supplementary file 1 — Supplementary Information. [file 41598_2021_4662_MOESM1_ESM.pdf]

Supplementary Information

AMIGO2 contained in cancer cell-derived small extracellular vesicles enhances the adhesion of liver endothelial cells to cancer cells

Runa Izutsu*, Mitsuhiko Osaki*†, Hideyuki Nemoto*, Maho Jingu*, Ryo Sasaki*, Yusuke Yoshioka‡, Takahiro Ochiya‡ & Futoshi Okada*†

*Division of Experimental Pathology, Faculty of Medicine, Tottori University, Yonago, Japan., †Chromosomal Engineering Research Center, Tottori University, Yonago, Tottori, Japan, ‡Department of Molecular and Cellular Medicine, Institute of Medical Science, Tokyo Medical University, Tokyo, Japan.

Corresponding Author: Mitsuhiko Osaki, Ph.D.

Division of Experimental Pathology, Faculty of Medicine, Tottori University

86 Nishi-cho, Yonago, Tottori 683-8503, Japan

Tel: +81-859-38-6242; Fax: +81-859-38-6240

E-mail: osamitsu@tottori-u.ac.jp

Supplementary Figure S1

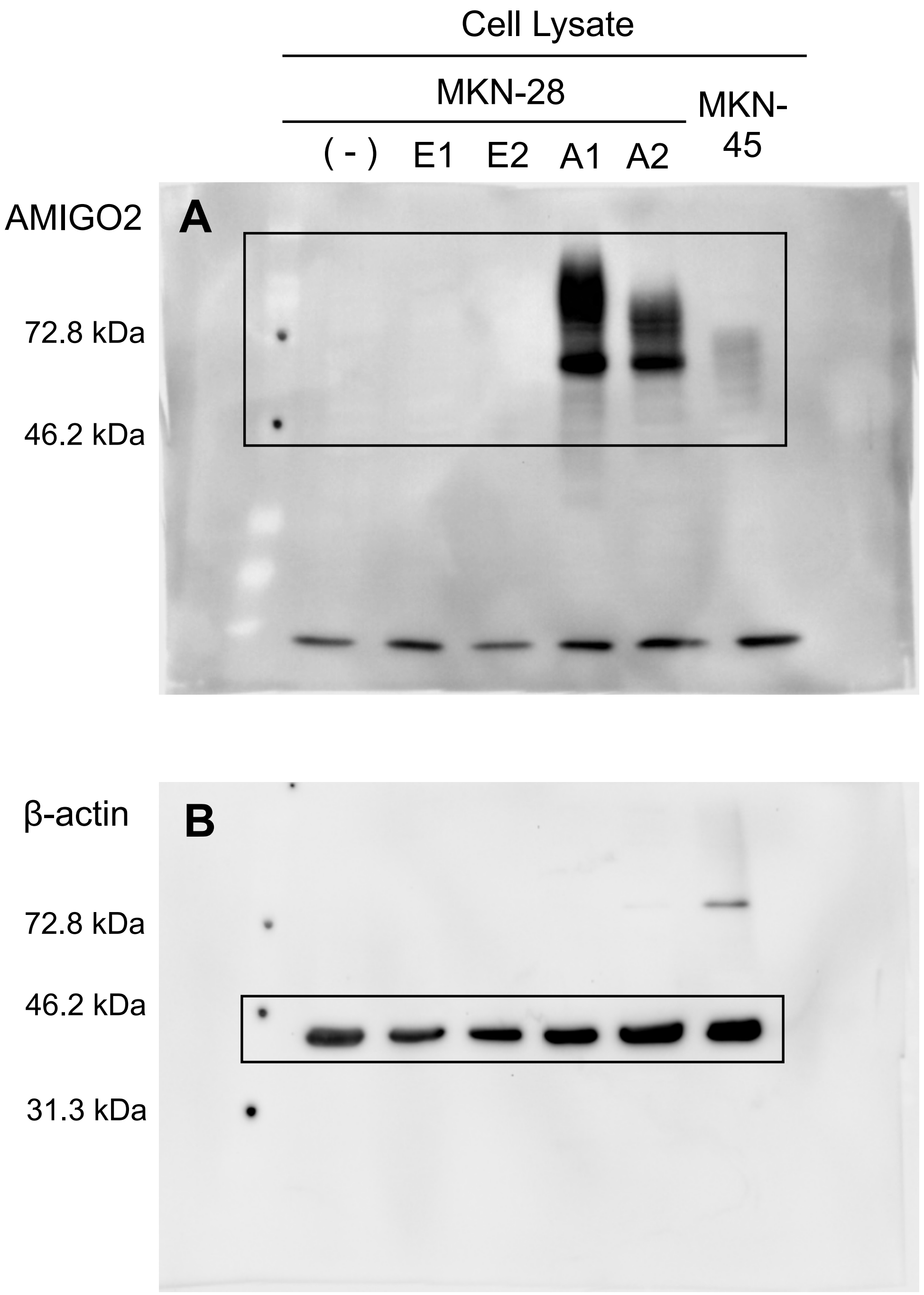

Supplementary Figure S1. Whole Western blots from Figure 1a

Full-length Western blots of AMIGO2 (A) and β-actin (B) in Figure 1a. Both blots represented unprocessed original image data, and the cropped area is shown in Figure 1a. AMIGO2 bands of membrane A was stripped by treatment with WB stripping Solution (05364-55, Nacalai Tesqe, Japan) and reproved with anti-β-actin antibody for confirmation of equivalence of the loading protein in each lane.

Supplementary Figure S2

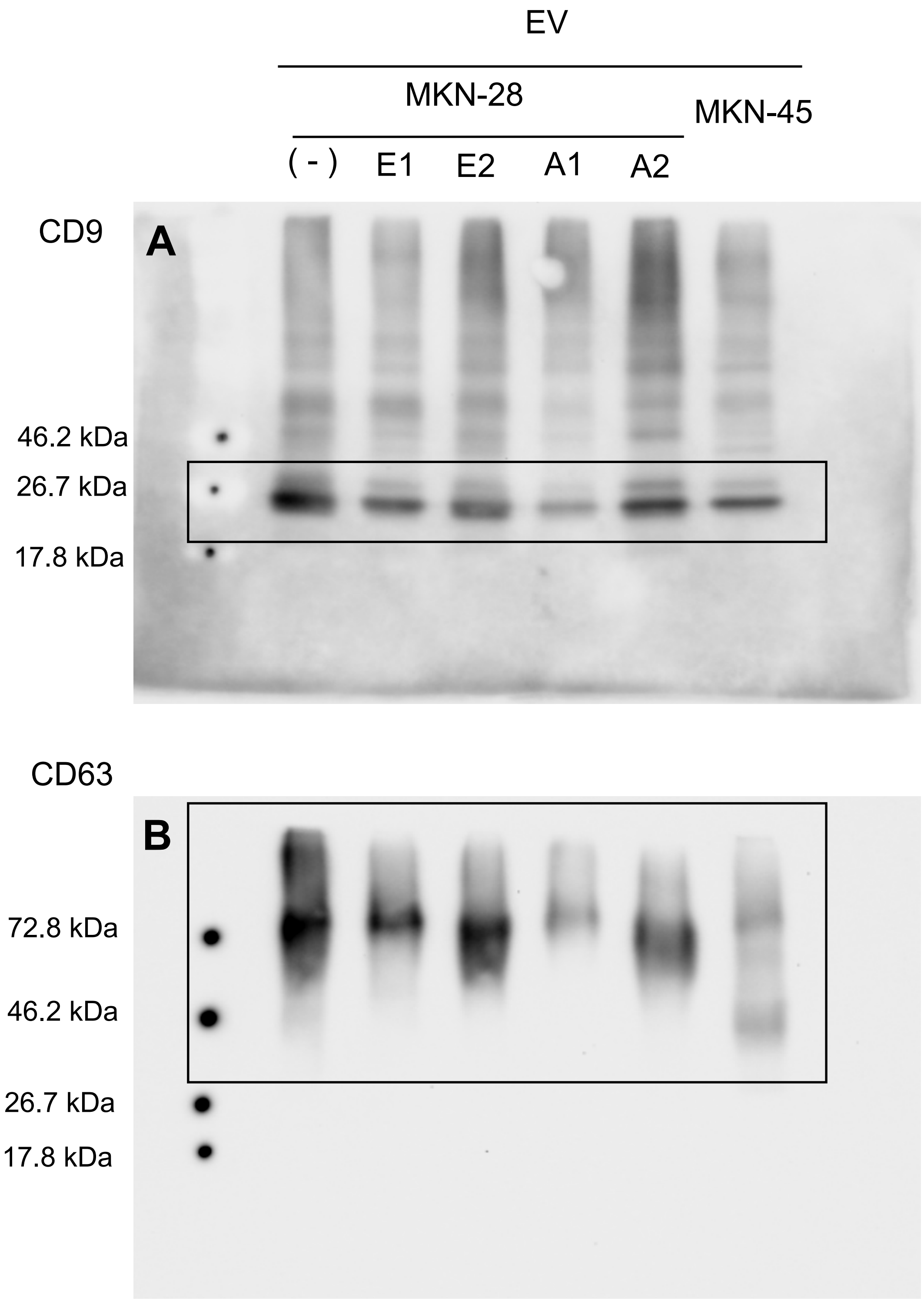

Supplementary Figure S2. Whole Western blots from Figure 1c

Whole gel images of Western blots of CD9 (A) and CD63 (B), which are typical EV markers in Figure 1c. Both blots represented unprocessed original image data, and the cropped area is shown in Figure 1c.

Supplementary Figure S3

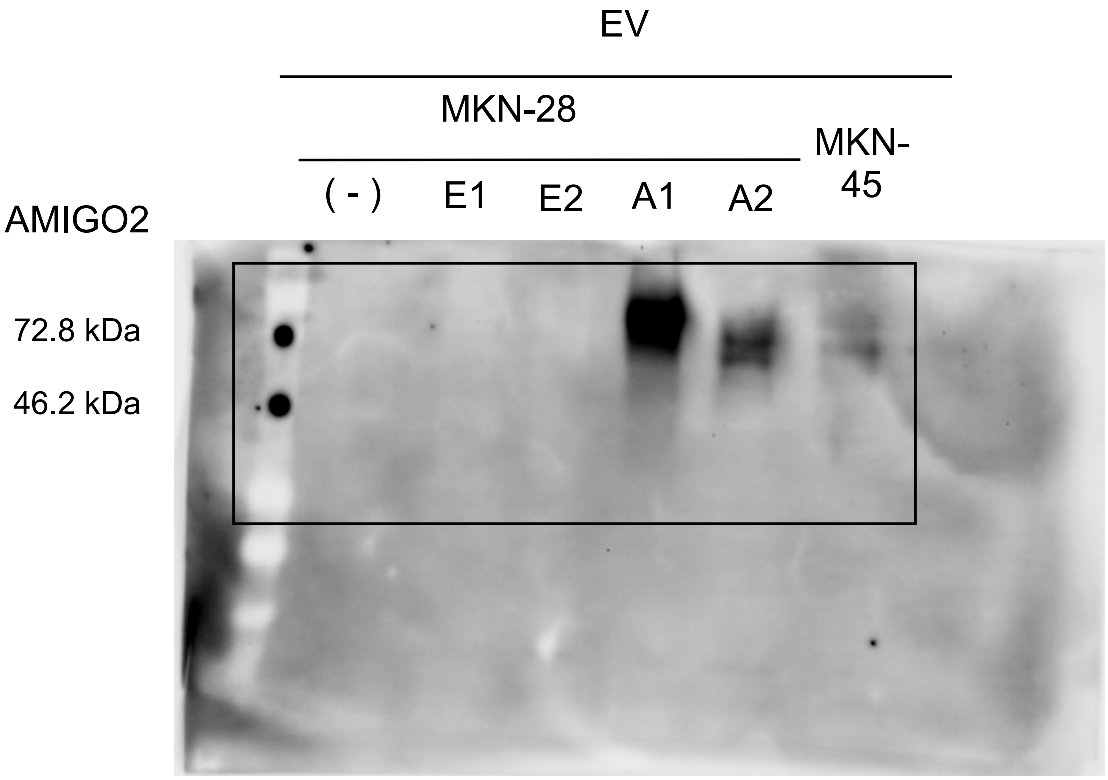

Supplementary Figure S3. Whole western blots from Figure 1d

Full-length Western blots of AMIGO2 in EVs obtained from cancer cells in Figure 1d. The membrane blots represented unprocessed original image data, and the cropped area is shown in Figure 1d.

Supplementary Figure S4

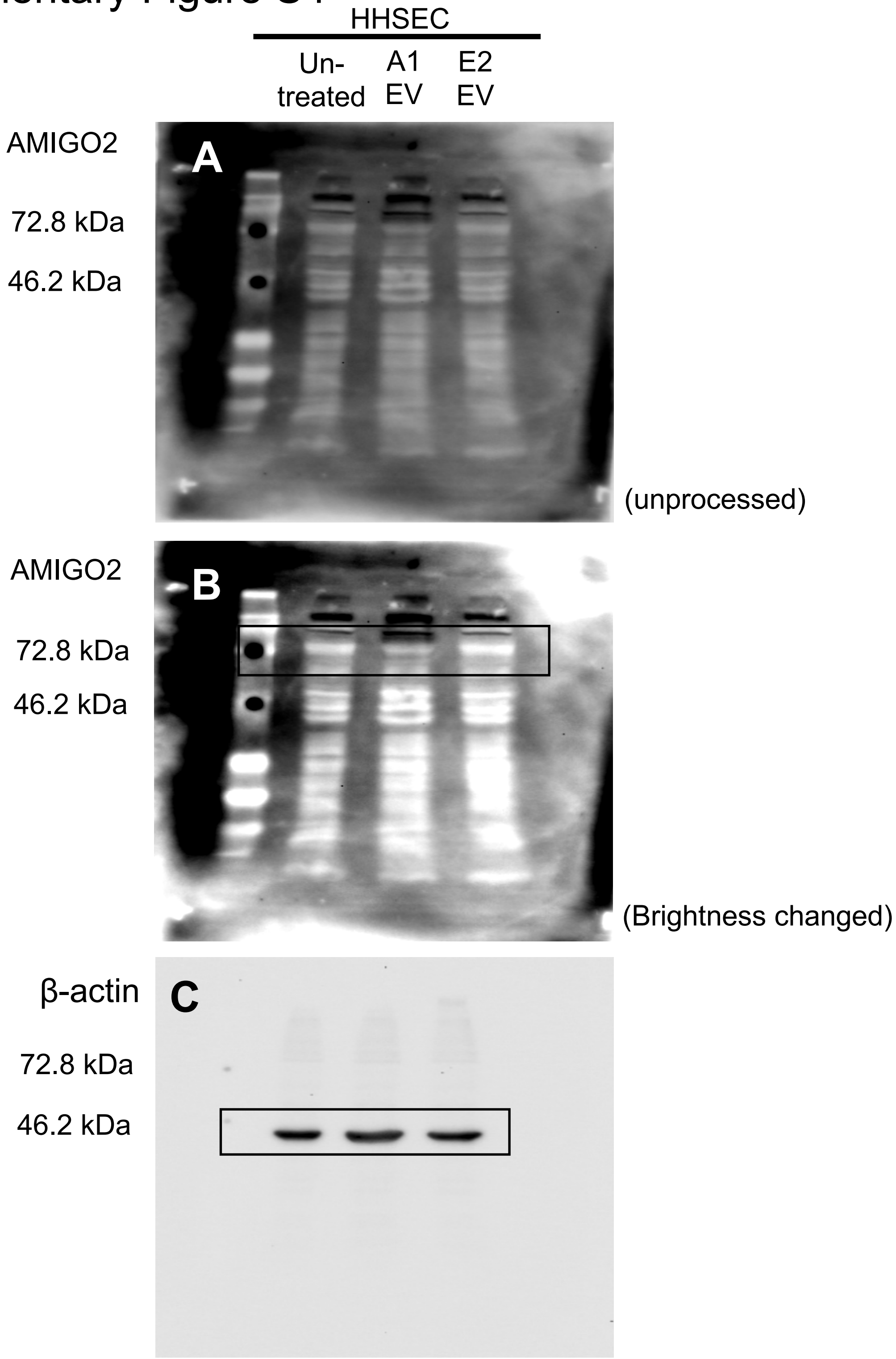

Supplementary Figure S4. Whole western blots from Figure 2c

Whole gel images for Western blots of AMIGO2 and β-actin in Figure 2c. The membrane A represented unprocessed original image data, and membrane B was changed brightness of whole membrane A using imageJ (version 1.53, National Institutes of Health, USA) and the cropped area is shown in Figure 2c. AMIGO2 bands of membrane B was stripped by treatment with WB stripping Solution (Nacalai Tesqe, Japan) and reproved with anti-β-actin antibody for confirmation of equivalence of the loading protein in each lane.

Supplementary Figure S5

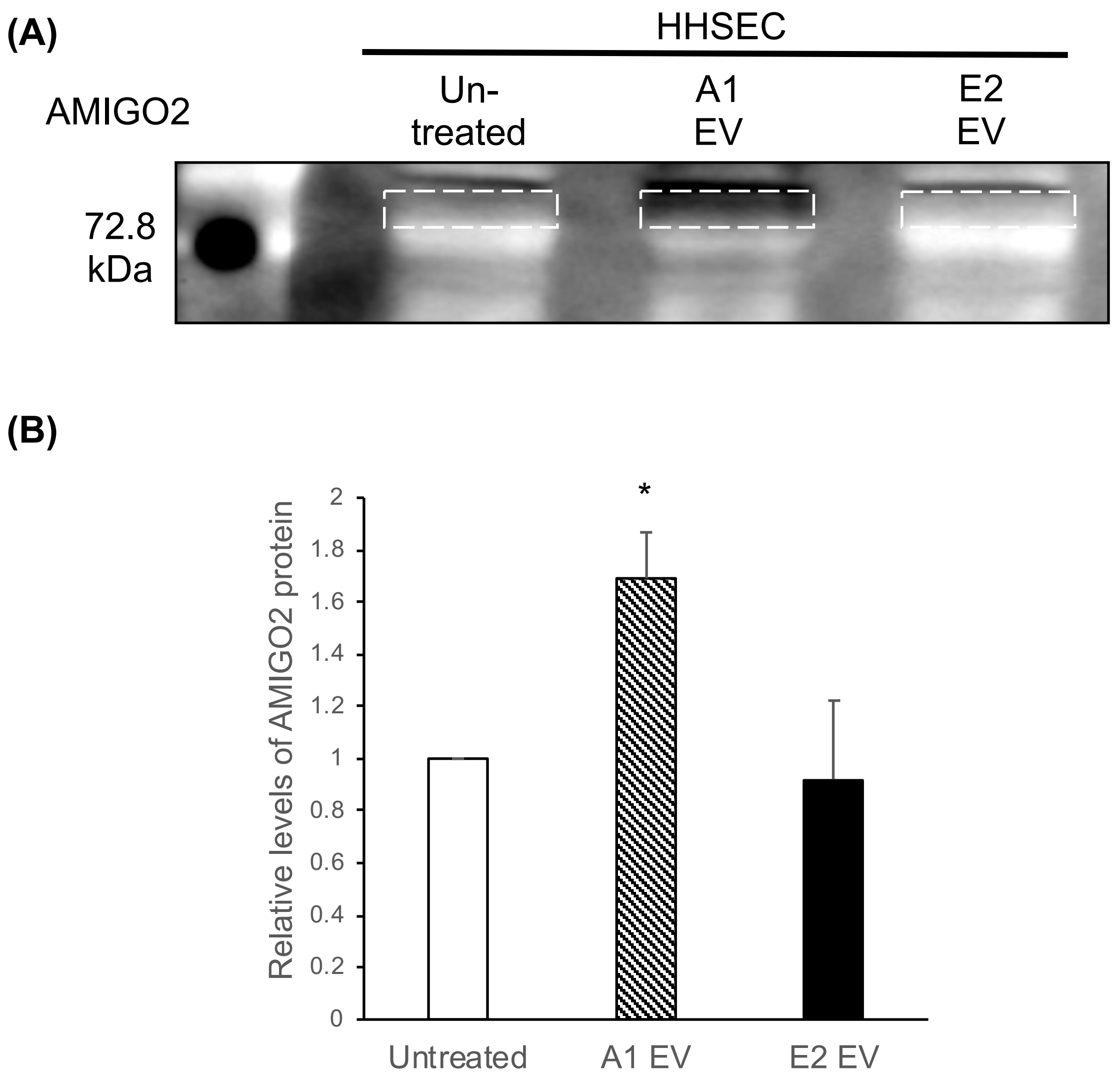

Supplementary Figure S5. Relative levels of AMIGO2 protein

(A) Western blot analysis showing increased AMIGO2 expression in A1 EV-treated HHSECs compared to untreated or E2 EV-treated cells.

(B) The relative protein expression level of AMIGO2 was quantified using ImageJ (version 1.53, National Institutes of Health, USA). Each bar represents the mean ± standard deviation of three independent experiments with similar results. *$P < 0.05$ (Student's t-test).

Supplementary Figure S6

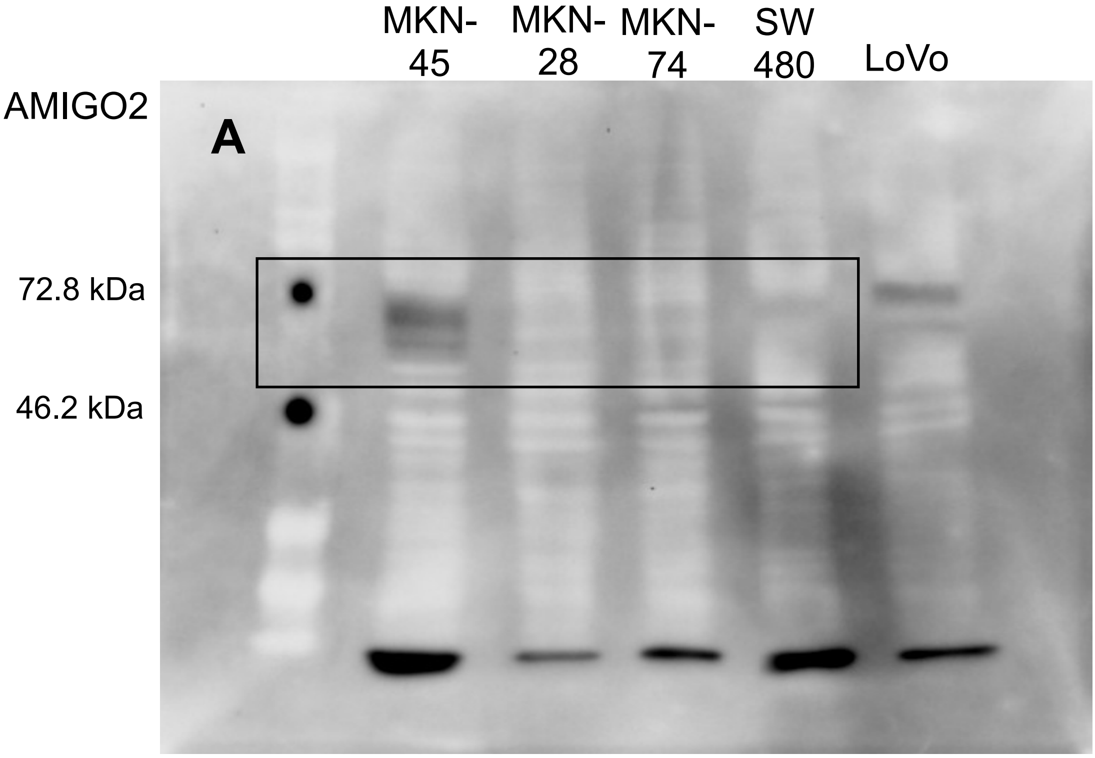

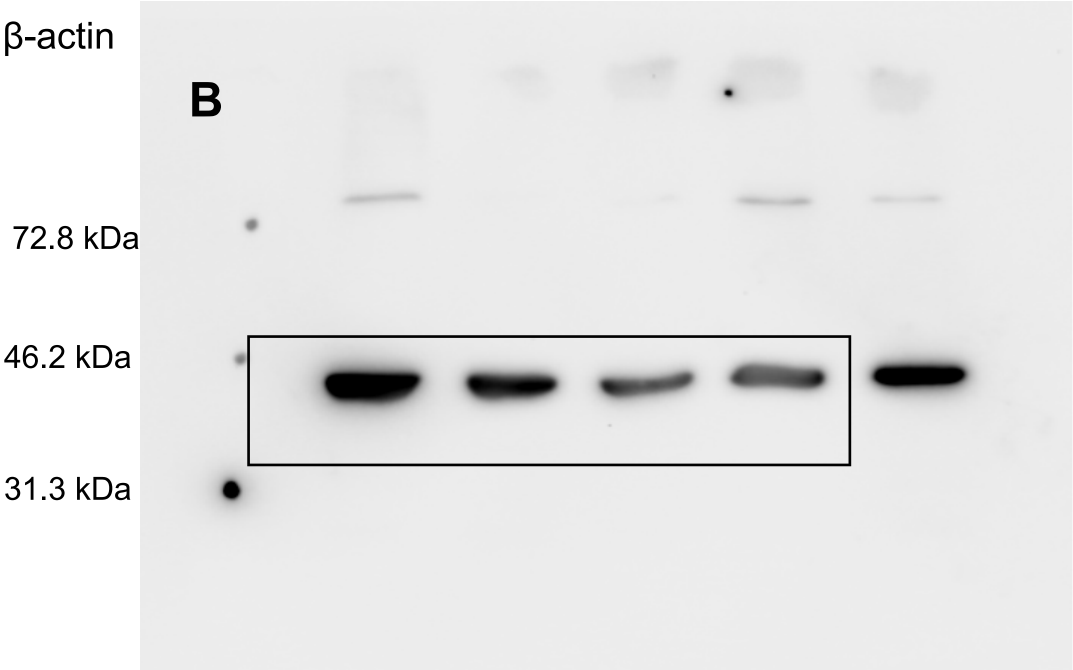

Supplementary Figure S6. Whole Western blots from Figure 3b

Full-length Western blots of AMIGO2 (A) in cancer cells and β-actin (B) in Figure 3b. Both blots represented unprocessed original image data, and the cropped area is shown in Figure 3b. AMIGO2 bands of membrane A was stripped by treatment with WB stripping Solution (Nacalai Tesqe, Japan) and reproved with anti-β-actin antibody for confirmation of equivalence of the loading protein in each lane.

Supplementary Figure S7

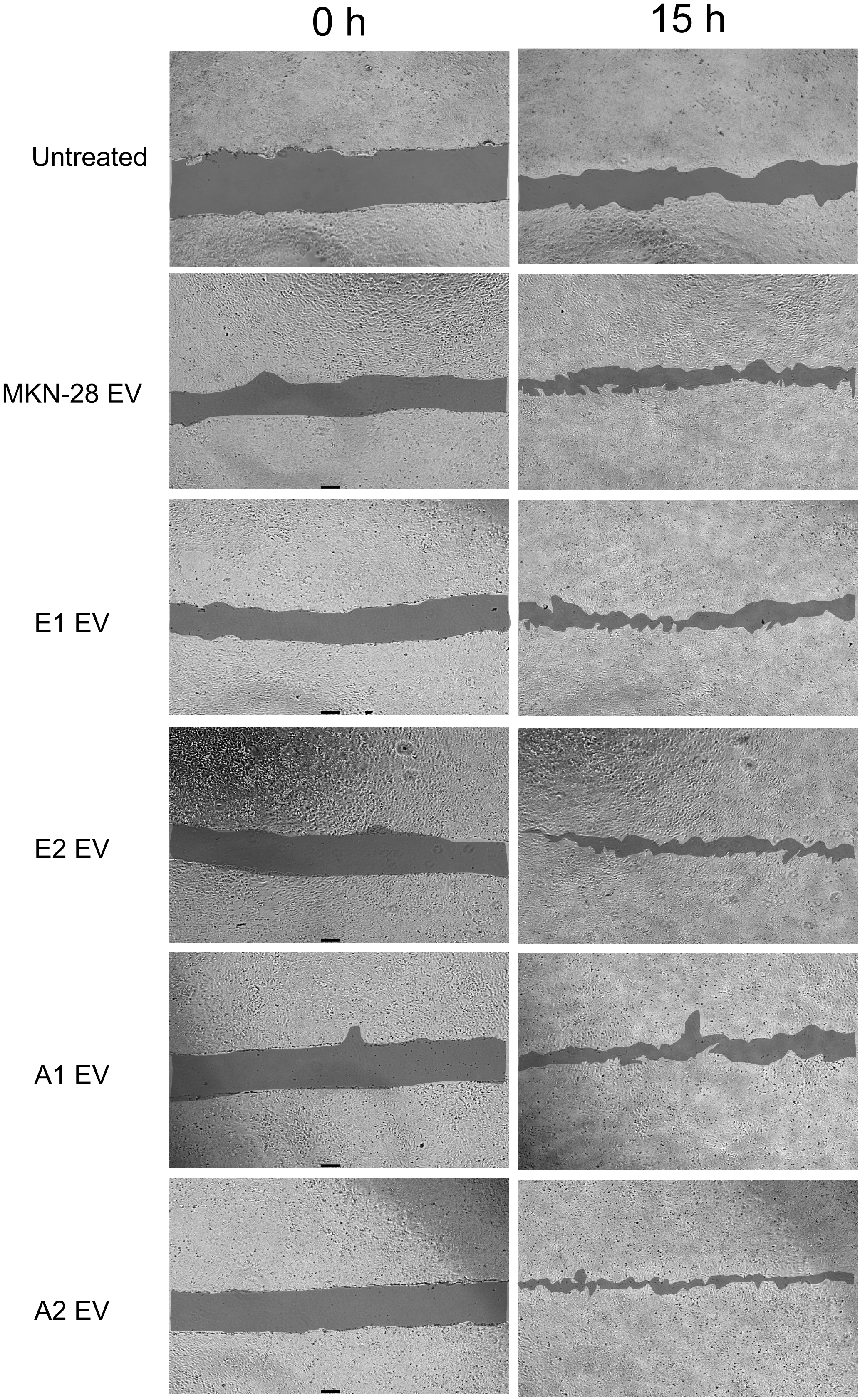

Supplementary Figure S7. Typical data of wound-healing assay.

At 0 h and 15 h after treatment of HHSECs with EVs, the scratched was observed using a phase-contrast microscope (BZ-X710, Keyence). Scratched area was quantified using ImageJ (version 1.53, National Institutes of Health, Bethesda, MD, USA).

Supplementary Figure S8

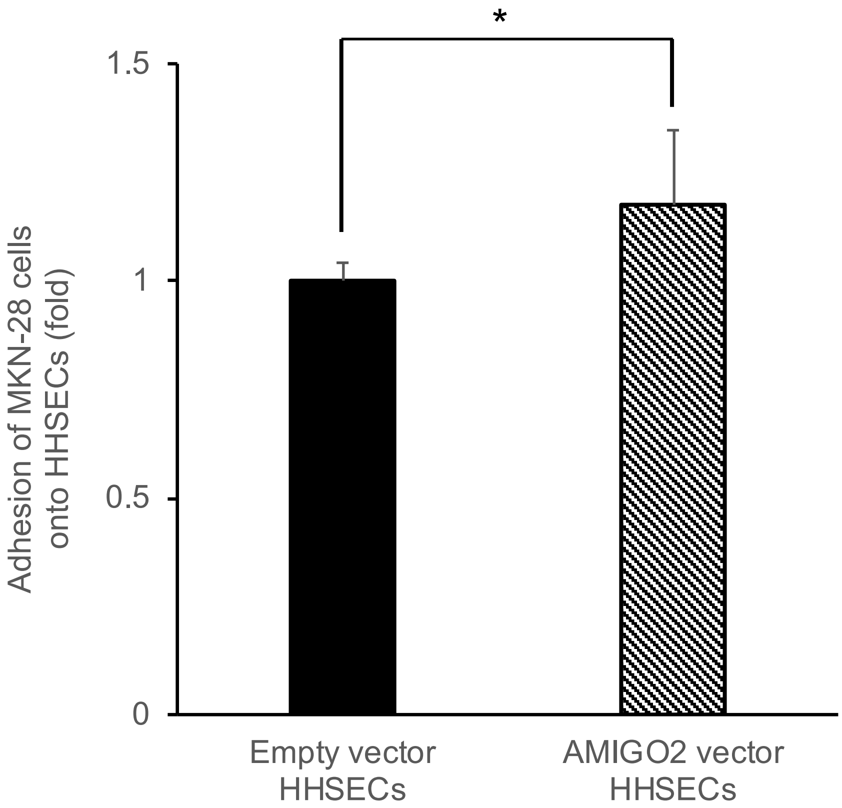

Supplementary Figure S8. Adhesion of MKN-28 cells onto HHSECs transfected with AMIGO2 plasmids.

HHSECs were transiently transfected with AMIGO2 or empty plasmids respectively. The adhesion of MKN-28 cells to HHSECs was increased compared to HHSECs transfected with empty plasmids. Each bar represents the mean ± standard deviation (n=5). *P < 0.05 (Student's t-test).

Supplementary Figure S9

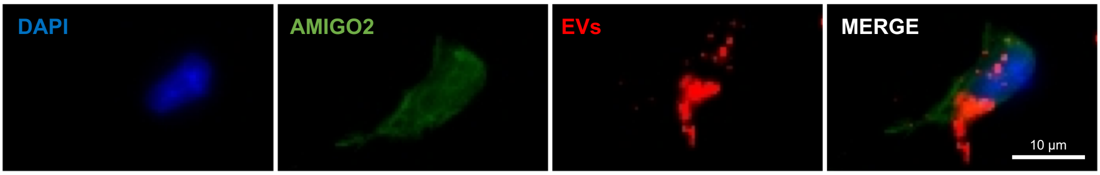

Supplementary Figure S9. Colocalization of AMIGO2 and EVs.

Colocalization of AMIGO2(green) and EVs(red) was observed in HHSECs treated with AMIGO2-containing EVs using microscopy (BZ-X710, Keyence).
